# Decentralized Online Simultaneous Localization and Mapping for Multi-Agent Systems

**DOI:** 10.3390/s18082612

**Published:** 2018-08-09

**Authors:** Andrés C. Jiménez, Vicente García-Díaz, Rubén González-Crespo, Sandro Bolaños

**Affiliations:** 1 Department of Engineering, Distrital University Francisco José de Caldas, 110221 Bogotá, Colombia; sbolanos@udistrital.edu.co; 2 Department of Electronic Engineering, Los Libertadores Foundation University, 110221 Bogotá, Colombia; 3 Department of Computer Sciences, University of Oviedo, Street San Francisco 1, 33003 Oviedo, Spain; garciavicente@uniovi.es; 4 Department of Computer Sciences, Universidad Internacional de La Rioja (UNIR), Avenida de la Paz 137, 26004 Logroño La Rioja, Spain; ruben.gonzalez@unir.net

**Keywords:** intelligent robots, mobile agents, multi agent systems, simultaneous localization and mapping, wireless sensor networks

## Abstract

Planning tasks performed by a robotic agent require previous access to a map of the environment and the position where the agent is located. This creates a problem when the agent is placed in a new environment. To solve it, the RA must execute the task known as Simultaneous Location and Mapping (SLAM) which locates the agent in the new environment while generating the map at the same time, geometrically or topologically. One of the big problems in SLAM is the amount of memory required for the RA to store the details of the environment map. In addition, environment data capture needs a robust processing unit to handle data representation, which in turn is reflected in a bigger RA unit with higher energy use and production costs. This article presents a design for a system capable of a decentralized implementation of SLAM that is based on the use of a system comprised of wireless agents capable of storing and distributing the map as it is being generated by the RA. The proposed system was validated in an environment with a surface area of 25 m${}^{2}$, in which it was capable of generating the topological map online, and without relying on external units connected to the system.

## 1. Introduction

Navigation in an unknown environment is one of the most difficult tasks for a robotic agent (RA) because it has to sacrifice autonomy for the increased difficulty of not knowing its location, the map, or the possible routes it could take to fulfill its objective. Currently, there are techniques for location and mapping (SLAM) that enable a single agent or a group of agents to capture information from their environment and generate the map by using sensors.

One of the most widely used types of sensors in SLAM is the laser telemetry sensor or LIDAR. LIDAR sensors measure distances by sending a pulse of infrared light or LASER to bounce off surrounding objects. The reflected light is then captured by a scanner and processed [1,2,3]. Other sensors used in SLAM beside LIDAR are monocular or stereo cameras that capture images from the environment. The images obtained are then processed to generate a three-dimensional map [4].

However, the use of both LIDAR sensors and cameras require a large amount of memory to process and represent the environment map, as they estimate the RA’s position through predictive and optimization techniques such as Bayesian filters, particle filters, and Kalman filters with normal distributions [5,6,7].

The proposed system uses wireless sensor network technology (WSN) [8], specifically the power radiation pattern produced by the wireless nodes in the network, known as Received Signal Strength Indication (RSSI) to reduce the processing requirements to estimate the RA’s location in an unknown environment. This parameter is used to triangulate the RA’s position in the environment, and requires that a minimum of three nodes in the network are in range to detect the RA, to be able to estimate its position by using an external processing unit [9].

Besides serving to obtain the RA’s location, the a priori placement of sensors enables the RA to generate a topological map of the terrain by characterizing each network node as a vertex in the map graph, reducing the complexity and the amount of memory required to process the RA’s location and to generate the map.

However, by requiring the WSN nodes to be positioned a priori and that the processing is performed by an external unit, this approach reduces the system’s autonomy. It also requires that each node is placed uniformly in the map, which increases deployment complexity in areas of difficult access.

This article presents a novel technique for SLAM that uses an RA to install static wireless agents (WA) in the environment, which form a multi-agent system (MAS) based on WSN principles to generate topological maps. For this purpose, we propose a differential RA which is tasked to program and install the WAs, equipped with distance sensors for obstacle detection, a digital compass to allow the RA to obtain its bearings, and encoders in all its motors to estimate the distance traveled in its displacement.

To begin the location process, the RA begins by creating a graph in which each node will represent an (x,y) position in $R^{2}$, and the connections between nodes will represent the continuity lines for the topological map. The graph will add new nodes as the RA detects new obstacles or crossings between its previous traveled routes, and the value of each new node will be determined by processing the odometry of the RA’s motors and the orientation provided by its digital compass.

Finally, the WA installation is performed when the RA scans its environment and is unable to detect another WA in its RSSI range. During this process, the graph is programmed in the WA, which receives constant updates while it remains within range of the RA. This allows the map to be continued by a new RA in the case the RA breaks down.

The technique introduced in this article presents three major contributions. Firstly, the system disposes of the requirement of prior installation of the WA used for obtaining the RA location. Secondly, it eliminates the dependency on an external unit to process data, enabling the RA to generate the map online. The third major contribution is that the MAS system is comprised of a single AR and multiple static robotic agents that store the routes. Unlike systems with multiple robotic agents (MARS), the proposed system only requires the use of a single RA, which reduces complexity by not needing to control the behavior of multiple mobile agents [10]. In addition, in the case the RA fails, the WAs contain the generated topological map which can be loaded in a new RA, resulting in increased autonomy and fault tolerance of the system.

The article is organized as follows: Section 2 makes a brief recap of WSN mobile applications to explain multi-agent SLAM techniques using WA. Section 3 explains the system design and the SLAM algorithm used. Section 4 includes the tests performed in a 2D asymmetrical environment with the RA. Finally, Section 5 presents the conclusions of this work.

## 2. Related Work

WSN systems are characterized by their modularity and easy maintenance. Each node is comprised of three main units: a sensor unit, a processing unit, and a data transmission unit, with the latter using, in most cases, the 802.11 wireless networking specification. WSN technology has been implemented in developing monitoring systems, Internet of things (IoT) applications, and security systems, among others [11,12,13,14].

WSN nodes’ mobility can be strong or weak, depending on their being used in static or dynamic environments. If the network is deployed in a static environment, the nodes are classified as having weak mobility, and in most cases they are pre-installed in the environment to reduce processing time and data transmission latency, and also to increase battery range. If the environment is dynamic or the nodes can move autonomously, nodes are classified as having strong mobility [15].

In robotics, RAs can be classified as strongly mobile when they are capable of moving in open or closed environments to perform navigation tasks such as planning, location, and mapping. Caballero et al. [16] proposed a probabilistic framework using a network of robotic systems modeled after a WSN to estimate the location of each node in the WSN using an RA and employing particle filters with Bayesian weight classification methods where the weigh is updated by weighing the RSSI parameter. However, this approach is incapable of building a map of the environment, for which it requires the network to be pre-installed in the environment.

The RSSI parameter has been widely used to obtain a location of RAs in a new environment by using triangulation techniques. Elfadil [17] designed a WSN by installing each node in a specific position, enabling the RA to estimate its position through triangulation of RSSI param, Time Difference of Arrival (TDOA), and Time of Arrival (ToA). This approach has been employed by using a symmetrical node grid to build the WSN, which allows obtaining the location of the RA in the environment and performing planning tasks, as proposed by Zhou et al. [9]. For these approaches to succeed, all nodes need to be installed and programmed with their location in the environment, and also requires that none of the nodes fail.

WSNs have also been used to perform SLAM tasks, where the WAs are used as landmarks, sending their information to the RA to enable it to obtain its current location in the environment, while the RA simultaneously begins building the map. The information processed by the RA is then shared with the nodes, which help reduce the cost of building the map by doing it cooperatively. Wong et al. [18] showed how to build the map using a WSN, applying the Joint Probabilistic Data Association technique (JPDA) to reduce uncertainty errors in data processing. This technique assesses the surveyed information in three moments, past, present, and future. However, it requires a lot of computing power to build the map. Wijesoma et al. [19] proposed the use of a Maximum Data Association (MDA) value to reduce the computational level of processing, allowing to build maps with mobile obstacles.

Several techniques have been proposed to eliminate the dependency on having WAs being installed a priori in the terrain. Ollero et al. [20] proposed the use of an Unmanned Aerial Vehicle (UAV) to position sensors in an environment with no communication infrastructure, to monitor disaster areas or places of difficult access. However, this work does not employ techniques to build maps or locate the agents of the system. Tuna et al. [21,22] proposed the use of MARS systems to deploy WAs using the role-based exploration approach (explorers and transmitters) [23]. The RAs in the role of transmitters send the information to the WA nodes deployed in the terrain, and the explorer RA nodes send information to the central unit. The role of the explorers is to evaluate the RSSI pattern to deploy the WAs to survey the environment for the search of people in disaster situations. To perform location and mapping tasks, explorer agents sense the environment and build an individual map, which is shared with other agents through the central unit and performing SLAM tasks cooperatively (CSLAM) [24]. However, this technique reduces system autonomy by depending on a central unit to organize the RAs and to generate the maps.

The present article contributes by introducing a new technique to deploy WAs in the environment to build topological maps in a decentralized way, by not depending on a central processing unit. To achieve this, it employs a single robotic agent to generate the map and locate the WAs. In the case the RA fails, the WAs still have the already obtained information with the RA location and map, allowing to share this information with a new RA introduced in the system or with external monitoring units.

## 3. Localization and Multi-Agent Mapping System

### 3.1. Agents Description

The multi-agent system is comprised of a single RA and multiple WAs. The RA is in charge of generating an online map of its environment while marking critical points to deploy the WAs. During the WA deployment process, the RA is in charge of programming each node with the map and its estimated location in the environment. After the deployment process, a WA can accept connections with external agents (EA), which can be monitor agents (MA) to control the generation of the map, or planning agents (PA) to generate routes for new RA’s.

This article uses an RA that contains five distance sensors for obstacle detection in the environment, enabling it to evade obstacles and select possible routes simultaneously. The RA also contains a digital compass that enables the RA to obtain its bearings, two motors with encoders to estimate the distance covered, a processing unit to control the movement and generate the map, and a wireless unit for communications and programming of the WAs.

**Figure 1:**
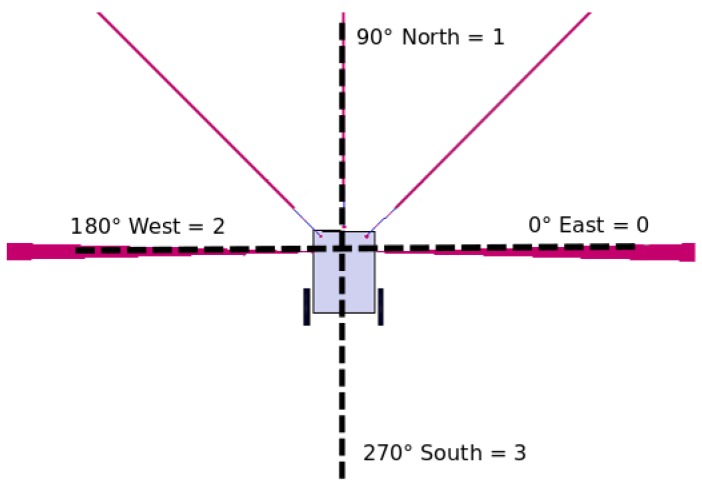
Identifiers assignation with respect to the cardinal directions of the RA.

The location system uses the digital compass and the motors odometry as its core. The digital compass locates the RA’s bearings to increment its position in the *X* or *Y* axes. Four cardinal positions are established to increment the traveled distance, in accordance to the value measured by the sensor, where 0-degrees is west, 90-degrees is north, 180-degrees is east, and 270-degrees is south. Each of the cardinal positions is stored in the RA and WAs memory by using an integer identifier, as shown in Figure 1.

The traveled distance is estimated through the motor encoders. However, the measurements obtained by the encoders can present errors derived from the varying sizes of wheel rims and the discrete resolution of readings, with these measurement errors being classified as systematic errors. For error-reduction purposes, the UMBmark model is implemented in the RA [25], based on the cinematic model described by the translation and rotation matrix of Equation (1).

(1) $$\left[\begin{array}{c} \dot{x}\\ \dot{y}\\ \dot{\theta } \end{array}\right]=\left[\begin{array}{cc} cos(\theta ) & 0\\ sin(\theta ) & 0\\ 0 & 1 \end{array}\right]\left[\begin{array}{c} \vec{r}\\ \theta \end{array}\right]+\left[\begin{array}{c} x\\ y\\ \phi \end{array}\right]$$

where:

$\dot{x}=$ New position *x* axis.

$\dot{y}=$ New position *y* axis.

$\dot{\theta }=$ New angle position.

$\vec{r}=$ Velocity vector of the robot.

θ= Rotation angle.

x= Actual position *x* axis.

y= Actual position *y* axis.

ϕ= Actual angle position.

**Figure 2:**
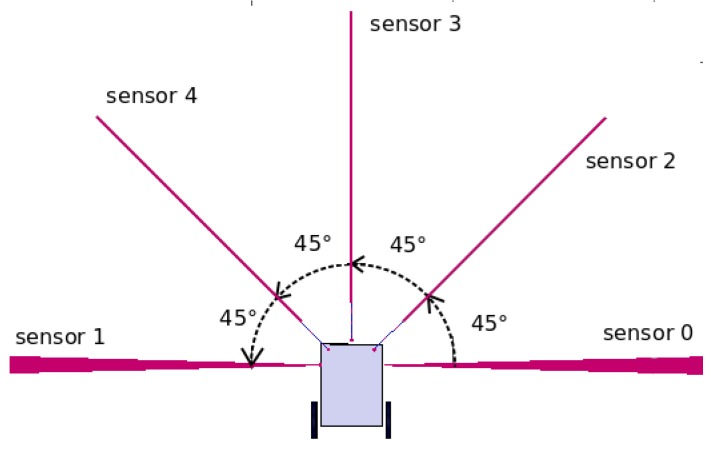
Placement and identification of the distance sensors of the RA.

To avoid collisions and detect possible routes, the RA uses distance sensors. Each of these sensors is separated by 45 degrees and are located at the front of the RA’s chassis, as shown in Figure 2. The sensors identified by numbers 2, 3, and 4 locate the obstacles that may impede forward motion, and the sensors identified as 0 and 1 detect lateral routes and obstacles.

### 3.2. Sensors Placement and Exchange of Information

The RA’s wireless unit is in charge of communications with the WAs. During the communications process, it validates if there are any new nodes to add to the units, with the goal of sharing the map with all the WAs in the network. It also uses RSSI to validate if there are any WAs in range. If there are no WAs in range, the RA programs a new node. Node programming consists of assigning it an SSID determined by the RA’s name and a consecutive installation number. The information is then stored in the node in a dynamic array to represent the map. This array contains four positions, as shown in Table 1. The first position indicates the node number in the topological map. Positions 2 and 3 in the array indicate the node’s position in the environment. The last position in the array contains the node to which it will be connected.

For the RA to be able to program a node, it needs to create a history record in its memory. The history is stored as a dynamic array in which the first four positions are the same as the ones in the WA nodes’ array, and adding a position to store the routes not traveled in the nodes (see Table 2). This position stores a string value of integer numbers to identify the pending cardinal position, separated by a period.

**Figure 3:**
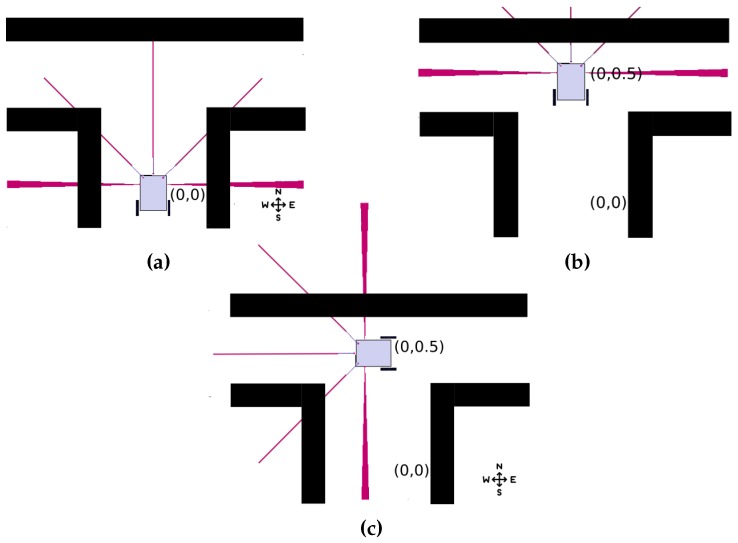
Robotic agent array update: (**a**) initial position; (**b**) adding new node; and (**c**) updating pending paths.

The RA updates its history according to the values received from sensors. If the forward sensors detect an obstacle that may cause a collision, or if the lateral sensors detect a space through which the RA may be able to navigate, these conditions cause the history record to be immediately updated. Figure 3a shows the RA in the initial position. At that moment, the first identifier is created with the initial conditions, which are the location (0,0) in $R^{2}$. It also detects the pending locations in location 1, which implies it can move to the north, relative to the value obtained by its digital compass. The agent validates the history to detect the possible routes, updates the history and moves. The RA keeps moving until the distance sensors detect a collision (see Figure 3b). This causes the history to be updated again with the location value processed through odometry and adds positions 0 and 2 as possible routes to the array. The agent reviews its history again, selects one of the two possible locations randomly, and executes a movement (Figure 3c), removing it from the list of pending locations (Table 3). This process is repeated until the RA detects a frontal collision and it has no pending routes in the RA history. Algorithm 1 shows the model of history update for the RA.

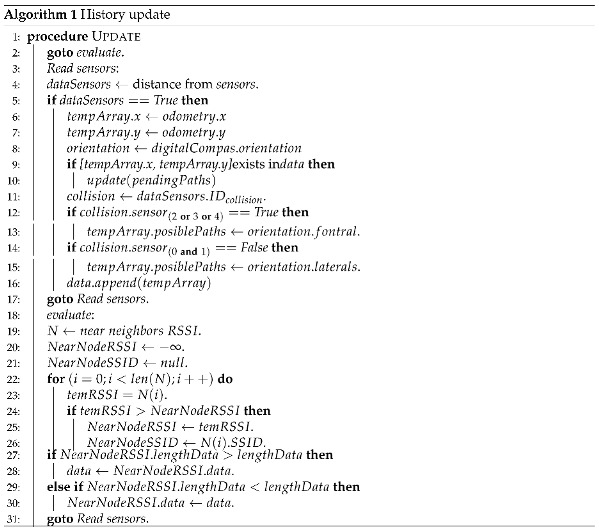



Communications between agents are decentralized, where the WA can perform the roles of Access Point or Client, while the RA performs the role of Client. This information exchange uses the multi-agent model of Xiong et al. [26], with the exception that the WAs will always be in communications range and they are in charge of sharing and generating the map working online with all the nodes in the network, while the RA only programs the WA with the highest RSSI value, reducing packet loss errors and the need to be continuously transmitting data to a central unit, which in turn increases battery autonomy for agents in the system.

When an RA enters a new environment, or a WA is deployed, the WA configures itself in client mode to search for other WAs within range. If it finds another WA, it begins an information exchange process, where the agent with the most stored information is the sender. In the case the information exchange takes place between an RA and WA, the RA stores the SSID of the WA transmitting the information, along with the number of nodes transmitted. If the client unit does not connect to another unit within a 5 s period, it automatically changes its configuration from Client to Access Point.

**Figure 4:**
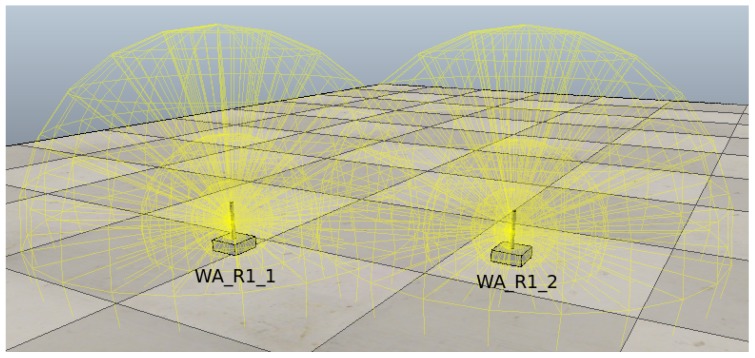
Information interchange between two agents.

Figure 4 shows two WAs that are about to initiate the information exchange process. The unit WA_R1_2 is initially set up to work in client mode and does not contain any stored SSIDs. The unit WA_R1_1 is in access point mode. The memory array of both units is represented in Table 4, with a total of six nodes in the unit WA_R1_2 and a total of four nodes in the unit WA_R1_1.

The unit WA_R1_2 connects to unit WA_R1_1 and initiates the information exchange process, detecting that two more nodes are stored in the unit WA_R1_2 as compared to unit WA_R1_1. These extra nodes are transmitted and stored along with the SSID WA_R1_2. Table 5 shows the arrays contained in both units after the information exchange.

To start the exchange of information, the processing unit is responsible for recognizing if a node is in the distance for read or write. If there are multiple nodes in the search range, the processing unit selects the closest node, while the RA checks if the node has routes for navigation. To fulfill the conditions, if the ID of the node is different from the last ID read, the RA takes the first path of the array and sends it to the position of navigated routes inside the array, thereby updating in the RA the identifier of the last node read. Otherwise, if the ID of the node is equal to the last one stored, then the reading of the node is ignored. Finally, if the identifier is different but the array has no pending paths, then the RA takes the path opposite to the position that the node has in its array and stores the ID of the wireless node. This process is shown in the Algorithm 2.

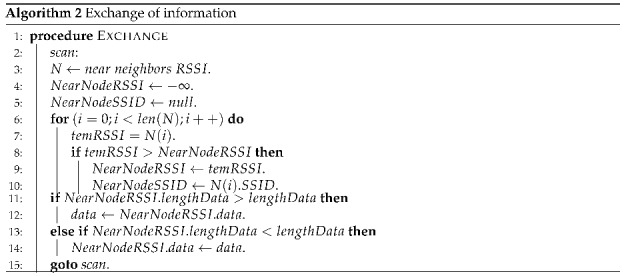



The exchange of information between a WA and a EA can be achieved as an exchange of information between two WAs, using Algorithm 2.

## 4. Validation and Analysis

Prior to the validation process, we need to model the use of the RSSI pattern. For this purpose, we assessed the behavior of the wireless module ESP8266, which is capable of being configured as client or access point. Its behavior was evaluated by sending 100 packets of 1024 bytes each, increasing the transmission distance until packet loss occurs.

**Figure 5:**
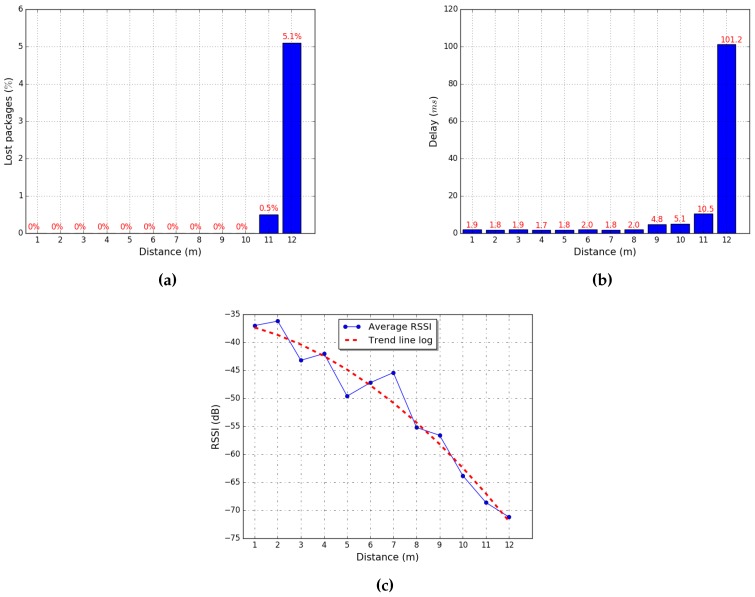
Evaluation of the wi-fi module ESP8266: (**a**) lost packages; (**b**) average delay; and (**c**) distance relation.

In the measurements obtained, we observed that packet loss occurs when the RSSI value is below −64 dB (see Figure 5a) and packets have a latency lower than 1.88 ms when the RSSI is below −50 dB (see Figure 5b). By measuring distance and evaluating the RSSI, we obtained a working range within 5 m (see Figure 5c).

**Figure 6:**
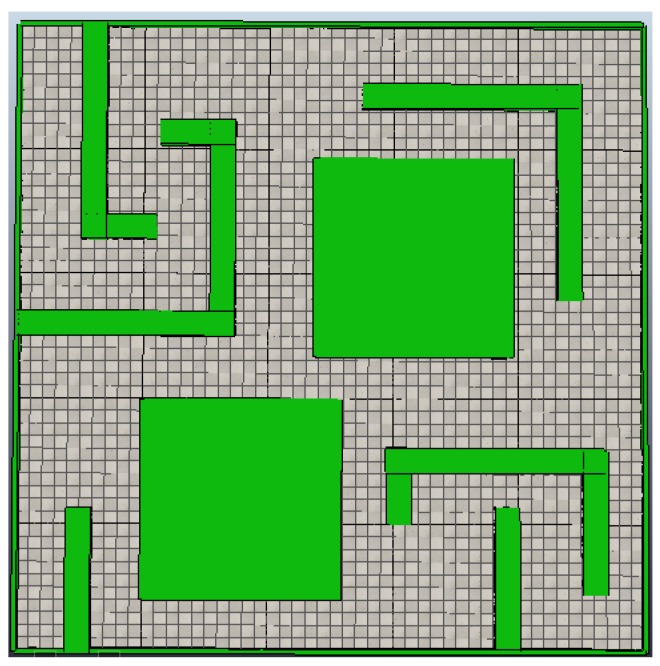
Test environment.

The validation for the proposed method for the online localization and mapping of unknown environments was performed through three experiments. The first experiment used the proposed method showing the capability of generating the map in a decentralized manner without the need for a priori WA deployment in the environment. The second experiment used a central unit for map generation; the time used to complete the map was then compared to the results of the first experiment. The third experiment was devised to assess the fault-tolerance capacity of the proposed system, compared to a centralized system. The three experiments were modeled in an environment with a surface area of 25 m × 25 m , as shown in Figure 6, in the V-Rep simulator, using a differential robotic agent with a constant velocity of 15 m/s . The wireless modules in the RA and WA agents in the system were modeled using an omnidirectional radiation pattern of 5 m, without taking into account signal loss due to obstacles.

**Figure 7:**
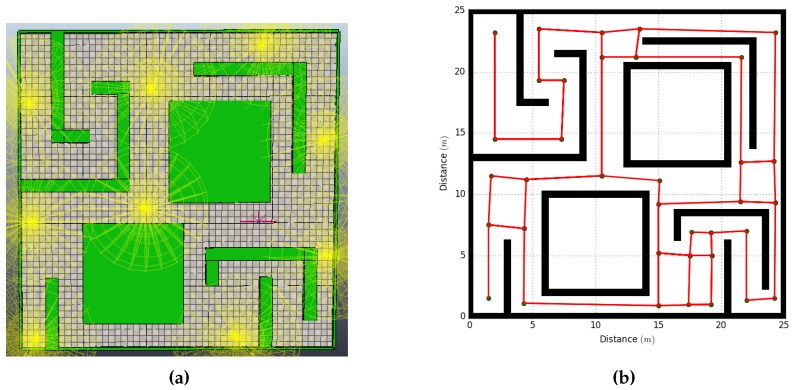
Results from the experiment using the proposed method: (**a**) WA nodes placement; and (**b**) Topological map.

The first experiment was able to build the topological map (Figure 7a) in 22 min and 20 s. During the SLAM process, the RA deployed a total of nine WAs to cover the full environment (Figure 7b). Table 6 shows the arrays from the agents after the test; the $x_{pos}$ and $y_{pos}$ values have an offset of −1.5 m, which correspond to the initial position on the environment.

**Figure 8:**
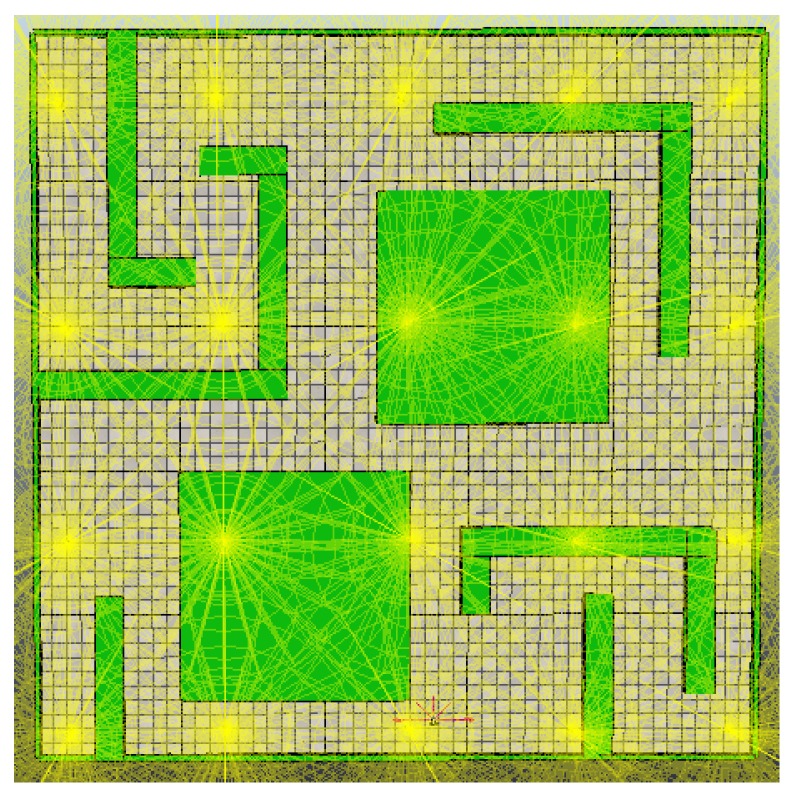
Placement of the wireless nodes in a centralized system.

The second experiment used a total of 16 preprogrammed nodes (Figure 8), deployed a priori in the environment, and reduced the time to generate the map to 16 min and 43 s, by performing location and mapping with the help of WSN nodes [26] and its central unit handling data processing. As shown in Table 7, the centralized unit has a better performance in timing, but it also increases the quantity of WAs in the environment.

**Figure 9:**
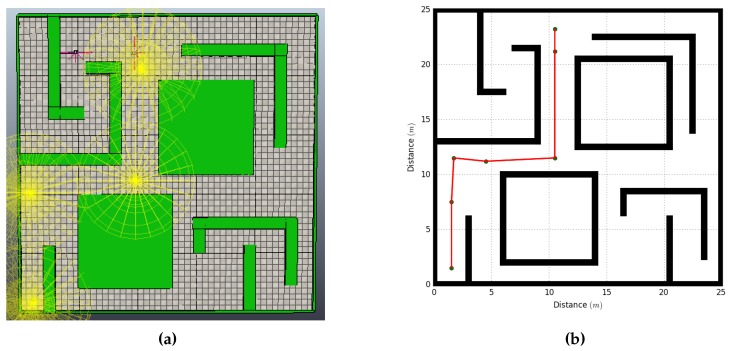
Failure injected to the RA: (**a**) WA nodes placement; and (**b**) topological map.

The third experiment introduced a communication error and a failure of the RA’s motors 7 min after the start of the experiment (Figure 9). When the WAs were unable to detect the RA in the system, they activated an alert message which could be received by an external unit or another RA. In this case, a second RA entered the system and updated its history according to the values stored in the existing nodes in the network (Table 8 and Table 9). The new RA was able to build the topological map in 18 min and 21 s, with a total of 25 min and 21 s. For modeling a failure in the centralized system, an error was introduced into the central communications unit, which impeded the system to complete the SLAM task.

The aforementioned experiments show the autonomous capabilities of the proposed system, highlighting the following items, which are a foundation to create an autonomous MAS [27,28]:

- **Modularity**: The WAs that compose the system do not need to be installed a priori in the environment for their functionality; they are installed by an RA during its navigation on the environment and can modify the information of the system network at any time. In addition, if an agent of the system fails, it can be replaced by another agent that presents the same characteristics, as was shown in the third experiment performed.
- **Decentralization**: This system does not require an external or central unit to perform control, location or mapping tasks. Even if the agents can interact between them to create the topological map and locate the RA in the environment, they do not need permanent communication.
- **Distributed processes**: Location and mapping tasks do not depend on any single unit, as information processing is performed by the RA and navigation data are stored and shared by the WA of the network.

## 5. Conclusions

This article presents a novel and decentralized multi-agent system capable of performing SLAM tasks by deploying wireless nodes in the environment through the use of an autonomous RA, avoiding the need for a central unit and the installation a priori of communication infrastructure. Three different experiments were performed in an unknown environment, showing that the proposed system is capable of generating a topological map online, by deploying static wireless agents and supported by the encoding of cardinal reference points and processing the odometry of the RA’s motors to establish its location, without requiring an external unit or the a priori deployment of network nodes.

The MAS decentralized system enables each WA to store an updated map with its own location stored in all the wireless nodes, by distributing the information across the whole network. This information-sharing process also makes the system capable of detecting failures such as an RA malfunction, or if any single WA fails, the RA is capable of deploying a new node in the area where it failed.

As future work, this system will be implemented to assess its behavior in rescue operations in emergency situations, as it is flexible and does not rely on any previously installed network to perform SLAM tasks, and can use other external agents to reduce the time to analyze the environment.

## Figures and Tables

**Table 1:** Dynamic array for the wireless agent.

Array-History WA			
$ID_{node}$	$x_{pos}$	$y_{pos}$	$LastID_{node}$

**Table 2:** Dynamic array for the robotic agent.

Array-History RA				
$ID_{node}$	$x_{pos}$	$y_{pos}$	$LastID_{node}$	Pending paths

**Table 3:** RA array update.

Array-History RA				
$ID_{node}$	$x_{pos}$	$y_{pos}$	$LastID_{\mathrm{node}}$	Pending Paths
0	0	0	0	-
1	0	0.5	0	0

**Table 4:** Initial arrays from the WA in Figure 4.

Array-WA_R1_1				Array-WA_R1_2			
Mode: Access Point				Mode: Client			
$ID_{node}$	$x_{pos}$	$y_{pos}$	$LastID_{\mathrm{node}}$	$ID_{node}$	$x_{pos}$	$y_{pos}$	$LastID_{\mathrm{node}}$
0	0	0	0	0	0	0	0
1	0	1	1	1	0	1	1
2	1	3	2	2	1	3	2
3	2	3	3	3	2	3	3
−	−	−	−	4	2	5	3
−	−	−	−	5	3	5	4

**Table 5:** Arrays from the WA in Figure 4 after the exchange of information.

Array-WA_R1_1				Array-WA_R1_2			
Mode: Client				Mode: Access Point			
$ID_{node}$	$x_{pos}$	$y_{pos}$	$LastID_{\mathrm{node}}$	$ID_{node}$	$x_{pos}$	$y_{pos}$	$LastID_{\mathrm{node}}$
0	0	0	0	0	0	0	0
1	0	1	1	1	0	1	1
2	1	3	2	2	1	3	2
3	2	3	3	3	2	3	3
4	2	5	3	4	2	5	3
5	3	5	4	5	3	5	4

**Table 6:** Arrays from Experiment 1 after the exchange of information.

Array-RA_1					Array-RA_1 (cont)				
Array-WA_R1_1 → Array-WA_R1_16					Array-WA_R1_1 → Array-WA_R1_16 (cont)				
$ID_{node}$	$x_{pos}$ **(m)**	$y_{pos}$ **(m)**	$LastID_{\mathrm{node}}$	Pending Paths	$ID_{node}$	$x_{pos}$ **(m)**	$y_{pos}$ **(m)**	$LastID_{\mathrm{node}}$	Pending Paths
0	0	0	0	−	18	20.5	−0.16	17	−
1	0	6	0	−	19	20.5	5.5	18	−
2	0.2	10	1	−	20	17.7	5.35	19	−
3	3	9.7	2	−	21	16.13	5.4	20	−
4	9	10	3	−	22	16	3.5	21	−
5	9	19.7	4	−	23	15.9	−0.5	22	−
6	9	21.7	5	−	24	17.7	−0.5	23	−
7	4	22	6	−	25	17.75	3.5	24	−
8	4	17.8	7	−	26	13.5	−0.6	23	−
9	6	17.8	8	−	27	2.8	−0.4	26	−
10	5.8	13	9	−	28	2.85	5.7	27	−
11	0.5	13	10	−	29	11.7	19.7	5	−
12	0.5	21.7	11	−	30	20.1	19.7	29	−
13	12	22	6	−	31	20.04	11.1	30	−
14	22.8	21.7	13	−	32	20	7.9	31	−
15	22.7	11.2	14	−	33	13.5	7.7	32	−
16	22.8	7.8	15	−	34	13.5	3.7	33	−
17	22.73	0	16	−	35	13.6	9.61	33	−

**Table 7:** Results from Experiments 1 and 2.

Decentralized		Centralized	
WA Used	Time (mm:ss)	WA Used	Time (mm:ss)
9	22:20	16	16:43

**Table 8:** Arrays transferred to the new RA from Experiment 3 after the injected failure.

Array-RA_2				
Array-WA_R1_1 → Array-WA_R1_4				
$ID_{node}$	$x_{pos}$ **(m)**	$y_{pos}$ **(m)**	$LastID_{\mathrm{node}}$	Pending Paths
0	0	0	0	−
1	0	6	0	0
2	0.2	10	1	−
3	3	9.7	2	3
4	9	10	3	0
5	9	19.7	4	0
6	9	21.7	5	0; 2

**Table 9:** Results from Experiment 3.

Decentralized		Centralized	
WA Used	Time (mm:ss)	WA Used	Time (mm:ss)
9	25:21	16	−−

## Abbreviations

The following abbreviations are used in this manuscript:

MAS	Multi-Agent System
MARS	Multi-Agent Robotic System
RA	Robotic Agent
SLAM	Simultaneous Localization and Mapping
RSSI	Received Signal Strength Indication
WA	Wireless Agent
WSN	Wireless Sensor Network

## References

[1] Elena L., Garc S., Barea R., Bergasa L.M., Molinos E.J., Arroyo R., Romera E., Pardo S. (2017). A Multi-Sensorial Simultaneous Localization and Mapping (SLAM) System for Low-Cost Micro Aerial Vehicles in GPS-Denied Environments. Sensors.

[2] Alismail H., Baker L.D., Browning B. Continuous Trajectory estimation for 3D SLAM from actuated lidar. Proceedings of the 2014 IEEE International Conference on Robotics and Automation (ICRA).

[3] Li J., Zhong R., Hu Q., Ai M. (2016). Feature-Based Laser Scan Matching and Its Application for Indoor Mapping. Sensors.

[4] Schleicher D., Bergasa L.M., Ocaña M., Barea R., López M.E. (2009). Real-time hierarchical outdoor slam based on stereovision and GPS fusion. IEEE Trans. Intell. Transp. Syst..

[5] Corff S.L., Fort G. (2013). Convergence of a Particle-based Approximation of the Block Online Expectation Maximization Algorithm. ACM Trans. Model. Comput. Simul..

[6] Corff S.L., Fort G. (2013). Supplement Paper to “Online Expectation Maximization Based Algorithms for Inference in Hidden Markov Models”. https://xxx.lanl.gov/abs/1108.4130.

[7] Dumont T., Le Corff S. (2014). Simultaneous localization and mapping in wireless sensor networks. Signal Process..

[8] Sahli N., Jabeura N., Badra M. (2015). Agent-based framework for sensor-to-sensor personalization. J. Comput. Syst. Sci. Int..

[9] Zhou N., Zhao X., Tan M. RSSI-based mobile robot navigation in grid-pattern wireless sensor network. Proceedings of the 2013 Chinese Automation Congress.

[10] Jiménez A.C., García-Díaz V., Bolaños S. (2018). A decentralized framework for multi-agent robotic systems. Sensors.

[11] Rashid B., Rehmani M.H. (2016). Applications of wireless sensor networks for urban areas: A survey. J. Netw. Comput. Appl..

[12] Lee J.S., Su Y.W., Shen C.C. A Comparative Study of Wireless Protocols: Bluetooth, UWB, ZigBee, and Wi-Fi. Proceedings of the IECON 2007-33rd Annual Conference of the IEEE Industrial Electronics Society.

[13] Li M., Zhuang M. An overview of Physical layers on wireless body area network. Proceedings of the International Conference on Anti-Counterfeiting, Security and Identification.

[14] Taboun M., Brennan R. (2017). An Embedded Multi-Agent Systems Based Industrial Wireless Sensor Network. Sensors.

[15] Silva R., Sa Silva J., Boavida F. (2014). Mobility in wireless sensor networks-Survey and proposal. Comput. Commun..

[16] Caballero F., Merino L., Gil P., Maza I., Ollero A. (2008). A probabilistic framework for entire WSN localization using a mobile robot. Robot. Autom. Syst..

[17] Elfadil O.M. Navigation algorithm for mobile robots using WSN. Proceedings of the 2013 International Conference on Computing, Electrical and Electronic Engineering (ICCEEE).

[18] Wong R., Xiao J., Member S., Joseph S.L., Shan Z. Data Association for Simultaneous Localization and Mapping in Robotic Wireless Sensor Networks. Proceedings of the 2010 IEEE/ASME International Conference on Advanced Intelligent Mechatronics.

[19] Wijesoma W.S., Perera L.D.L., Adams M.D. (2006). Toward multidimensional assignment data association in robot localization and mapping. IEEE Trans. Robot..

[20] Ollero A., Marron P.J., Bernard M., Lepley J., La Civita M., Van Hoesel L., De Andrés E. AWARE: Platform for autonomous self-deploying and operation of wireless sensor-actuator networks cooperating with unmanned AeRial vehiclEs. Proceedings of the SSRR 2007-IEEE International Workshop on Safety, Security and Rescue Robotics Proceedings.

[21] Tuna G., Gungor V.C., Gulez K. (2014). An autonomous wireless sensor network deployment system using mobile robots for human existence detection in case of disasters. Ad Hoc Netw..

[22] Tuna G., Gungor V.C., Potirakis S.M., Zeadally S. (2015). Wireless sensor network-based communication for cooperative simultaneous localization and mapping. Comput. Electr. Eng..

[23] De Hoog J., Cameron S., Visser A. Role-based autonomous multi-robot exploration. Proceedings of the 2009 Computation World: Future Computing, Service Computation, Cognitive, Adaptive, Content, Patterns.

[24] Moratuwage M.D.P., Wijesoma W.S., Kalyan B., Dong J.F., Namal Senarathne P.G.C., Hover F.S., Patrikalakis N.M. Collaborative multi-vehicle localization and mapping in marine environments. Proceedings of the OCEANS’10 IEEE Sydney.

[25] Ruan X., Li Y., Zhu X. Kinematic parameter calibration of two-wheeled robot. Proceedings of the 2012 IEEE International Conference on Mechatronics and Automation.

[26] Xiong J., Qin Q., Zeng K. A Distance Measurement Wireless Localization Correction Algorithm Based on RSSI. Proceedings of the 2014 Seventh International Symposium on Computational Intelligence and Design.

[27] Thrun S., Bücken A., Burgard W., Fox D., Fröhlinghaus T., Hennig D., Hofmann T., Krell M., Schimdt T., Kortenkamp D., Bonasso R.P., Murphy R. (1997). Map Learning and High-Speed Navigation in RHINO. Artificial Intelligence and Mobile Robots.

[28] Ota J. (2006). Multi-agent robot systems as distributed autonomous systems. Adv. Eng. Inf..

